# Graphical representation of ribosomal RNA probe accessibility data using ARB software package

**DOI:** 10.1186/1471-2105-6-61

**Published:** 2005-03-21

**Authors:** Yadhu Kumar, Ralf Westram, Sebastian Behrens, Bernhard Fuchs, Frank Oliver Glöckner, Rudolf Amann, Harald Meier, Wolfgang Ludwig

**Affiliations:** 1 Lehrstuhl für Mikrobiologie, Technische Universität München, D-85350 Freising Germany; 2 Max Plank Institute for Marine Microbiology, D-28359 Bremen, Germany; 3 International University Bremen, D-28759 Bremen, Germany; 4 Lehrstuhl für Rechnertechnik und Rechnerorganisation, Technische Universität München, D-85748 Garching, Germany

## Abstract

**Background:**

Taxon specific hybridization probes in combination with a variety of commonly used hybridization formats nowadays are standard tools in microbial identification. A frequently applied technology, fluorescence *in situ *hybridization (FISH), besides single cell identification, allows the localization and functional studies of the microbial community composition. Careful *in silico *design and evaluation of potential oligonucleotide probe targets is therefore crucial for performing successful hybridization experiments.

**Results:**

The PROBE Design tools of the ARB software package take into consideration several criteria such as number, position and quality of diagnostic sequence differences while designing oligonucleotide probes. Additionally, new visualization tools were developed to enable the user to easily examine further sequence associated criteria such as higher order structure, conservation, G+C content, transition-transversion profiles and *in situ *target accessibility patterns. The different types of sequence associated information (SAI) can be visualized by user defined background colors within the ARB primary and secondary structure editors as well as in the PROBE Match tool.

**Conclusion:**

Using this tool, *in silico *probe design and evaluation can be performed with respect to *in situ *probe accessibility data. The evaluation of proposed probe targets with respect to higher-order rRNA structure is of importance for successful design and performance of *in situ *hybridization experiments. The entire ARB software package along with the probe accessibility data is available from the ARB home page .

## Background

The introduction and use of comparative sequence analysis of appropriate marker genes as a powerful tool in taxonomy has substantially contributed to the rapid growth of molecular sequence databases such as EMBL [1], GenBank [2], and ribosomal RNA (rRNA) databases [3-5]. Evidently, molecular phylogenetic analyses have greatly influenced the restructuring of systematics especially in the case of prokaryotes. Nowadays, identification and classification at the species and higher taxonomic levels mainly relies on a genotypic approach, typically involving an analysis of small, and to a lesser extent, large ribosomal RNA gene (rRNA) structures. The backbone of the current taxonomy of the prokaryotes is almost exclusively based upon a phylogenetic network derived from comparative sequence analysis of the small subunit rRNAs and respective phylogenetic marker genes [6]. As 'living fossils', these molecules at least roughly reflect the evolutionary history of the respective organisms. The mosaic-like primary structures comprising highly variable to highly conserved or invariant regions provide diagnostic information for different levels of phylogenetic relationship. Consequently, this information can be used to identify oligonucleotide target regions unique to phylogenetic entities, for use as taxon-specific hybridization probes or PCR primers. Depending on the target site such oligonucleotide probes or probe combinations can be designed for phylogenetic groupings as diverse as bacterial species or an entire phylum.

Ever since the fluorescence *in situ *hybridization (FISH) technique became an integral part of the rRNA approach to microbial ecology and evolution [7], rRNA-targeted oligonucleotide probes have evolved into a widely used tool for the direct, cultivation-independent identification and enumeration of individual microbial cells or specific groups of bacteria in simple to complex natural environments. In this regard, a good probe design and careful further evaluation *in silico *plays a crucial role to ensure sensitivity and specificity of a potential probe in its practical application. Besides uniqueness of the target sequence, number, character and position of diagnostic residues, comprehensiveness with respect to the inclusion of members of the desired target group (taxon) and exclusion of non-members along with a target molecule or region accessibility in the real hybridization experiment, have to be taken into consideration. Recently, data on *in situ *accessibility of rRNA targets in several microorganisms have become available [8-11].

Since biology is a highly visual science, there is a general demand for tools to visualise the variety of biological knowledge as diagrams, illustrations, two-dimensional and three-dimensional reconstructions, and other types of graphical formats. Hence, the visualization of molecular data in an interactive and intuitive graphical user interface ideally will serve as third eye for a molecular biologist. In this paper, we describe how the ARB software package [3] provides a workbench for designing, evaluation and visualization of oligonucleotide probes in more intuitive way, using interactive graphical user interface to visually examine characteristics and criteria of target regions.

## Implementation

### Sequence data

Periodically retrieved raw gene data comprising small subunit rRNA from public databases such as EBI [1], Genbank [2], the RDP[4], and the sequence data determined in our laboratory and other partner groups are imported into the ARB database, processed according to a variety of criteria and finally provided as curated databases at the ARB projects web-site [13]. The current public release of small subunit rRNA database [3] containing only complete sequences was taken for designing, evaluation and visualization of probes and targets, respectively. Partial sequences are avoided as they greatly limit the probe design by reducing the number of potential target regions and also give no hint about the specificity of existing probes that target to non-sequenced regions of the respective rRNAs.

### The positional tree (PT) server

The PT-Server [3] is a suffix tree server implemented in the ARB software which is used for indexing all sequence data represented in the underlying ARB sequence database. Once established, the particular PT-Server allows rapid and exact searching for target regions with respect to sequence identity or uniqueness.

### Probe design and probe match

**Figure 1:**
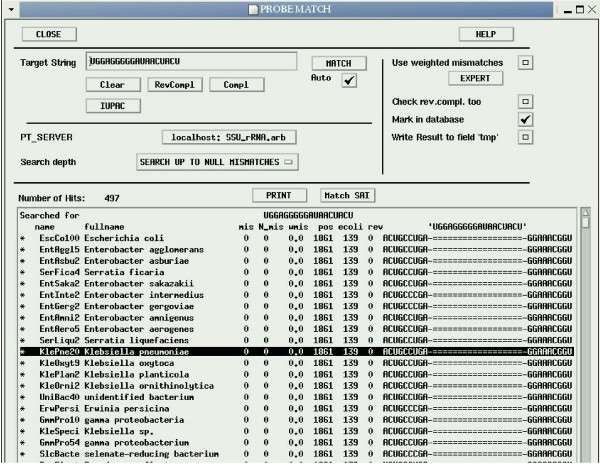
**Probe match window**. Probe Match Window showing results of *in silico *evaluation of the probe candidate (5'-UGGAGGGGGAUAACUACU-3') designed as part of a probe combination for the enterobacteria group. It hits 497 members of the group containing 947 species. Additional information such as number of mismatches, *E. coli *position, region up- and down-stream of the probe target in the actual nucleotide sequence along with the local alignment are also shown.

Probe design is carried out using the PROBE Design tool (PDT) of ARB software involving following steps:

1. The user selects the target group or a species of interest.

2. The parameters such as size of the probe and the probable physico-chemical characteristics like %GC content, melting temperature (T_m_) according to the 4°C GC, 2°C AT rule [14], and self-complementarity (hair-pin bonds) are specified. Optionally, a range of allowed target positions within the sequence alignment of the respective database can be defined.

3. Potential probe candidates are searched involving the respective PT-Server. Both, target and probe sequence are displayed in a result list. Ranking within this list follows estimated probe quality according to criteria defined for probe design such as number, character and position of diagnostic residues, coverage of the target group, physicochemical demands, which are displayed in separate probe results window along with relevant information.

4. Once the user selects the desired probe in the result list, it can be evaluated against the entire database by using the PROBE Match tool (PMT) of ARB. PMT, by default evaluates the targets for the sequence (strand) stored in the database. Optionally, the complementary sequence (opposite strand) can be evaluated as well. Members of the target group are displayed in a separate PROBE Match window along with other information such as number of mismatches, weighted mismatches, *E. coli *positions, reverse complementarity and local alignment of probe targets (Figure 1).

## Results and discussion

As the demand for oligonucleotide probes that can identify and quantify bacteria by nucleic acid hybridization is permanently increasing, *in silico *evaluation and visualization of such probes and targets are necessary, particularly, when used for FISH experiments. Target accessibility is among the crucial criteria to be evaluated with respect to experimental success of the respective probe based identification and detection system [7-12]. To facilitate this evaluation procedure, new functionalities were added to the ARB software package providing a more intuitive graphical environment. As an example, oligonucleotide probes were designed for the enterobacteria group represented by 947 database entries. The 5'-UGGAGGGGGAUAACUACU-3' probe was selected from the list of potential probes and evaluated against the background of the full dataset of complete and partial small subunit rRNA sequences. The selected probe perfectly matches the respective target of 497 members of the enterobacteria group (Figure 1). The same probe has been visualized in all the screenshots presented in the paper.

Although a phylogenetic probe is primarily judged in terms of its taxonomic range to identify the members of its intended target taxon to the exclusion of non-target bacteria, for a practical consideration it must also fulfil certain other criteria with respect to its applicability depending on the particular hybridization format. In case of the fluorescence *in situ *hybridization approach the results of the accessibility studies conducted by Fuchs and co-workers on the 16S and 23S rRNA of *Escherichia coli *and other organisms are among such criteria. They showed that some regions of *E. coli *ribosome are virtually inaccessible for oligonucleotide probes when FISH is performed [8,9]. They proposed a color code assigned to six intensity classes of *in situ *hybridization signals. Within the ARB program, these classes are coded in respective SAIs (so called Sequence Associated Information) and optionally visualized as background colors of the sequences in primary structure (ARB_Edit4), secondary structure (SEC_Edit), and probe visualization windows (PROBE Match) of ARB. All the displays produced by the ARB software are interconnected and any changes in one window are automatically updated in other windows as well. Simultaneous visualization and evaluation of oligonucleotide probes in different levels allows the user to look carefully and closely into the proposed probe candidates *in silico*, before carrying out further *in situ *or *in vivo *studies. More importantly, the user can perform a variety of sequence related operations such as importing sequence data, aligning, treeing, designing, evaluation and visualization of probes, performing statistical calculations and many other functions using interoperating and user friendly tools controlled from a common graphical platform within the ARB software package.

Visualization of potential probe candidates and the sequence associated information (SAI) such as higher order structure, conservation, G+C content, transition-transversion profiles and *in situ *target accessibility patterns, is possible at three different levels: the local alignment (PROBE Match tool), global alignment (ARB Primary Structure Editor) and secondary structure levels (Secondary Structure Editor).

### Visualization of SAI in probe match window

**Figure 2:**
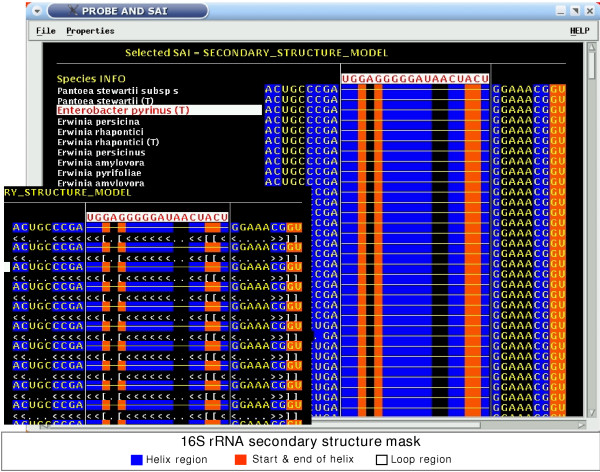
Screenshot displaying 16S rRNA secondary structure model where helix region is colored in blue, starting and ending positions of helix halves are in red and bases without background represent commonly non base paired positions.

**Figure 3:**
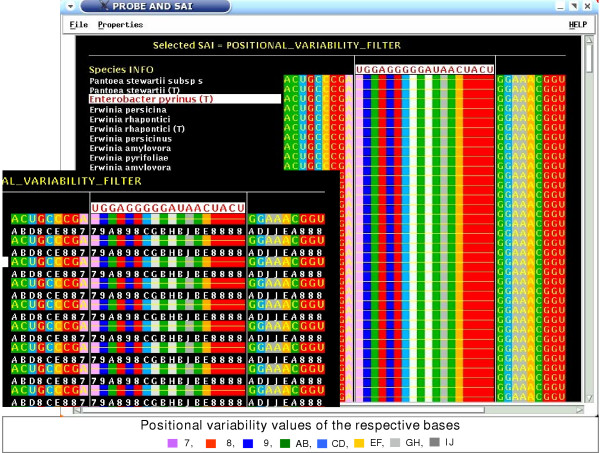
Screenshot showing positional variability filter generated by the respective ARB tool in various background colors (light purple – 7; red – 8; dark blue – 9; green – AB; blue – CD; yellow – EF; light grey – GH; grey – IJ; increasing numbers followed by the alphabetical order of letters indicate increasing degree of sequence conservation).

**Figure 4:**
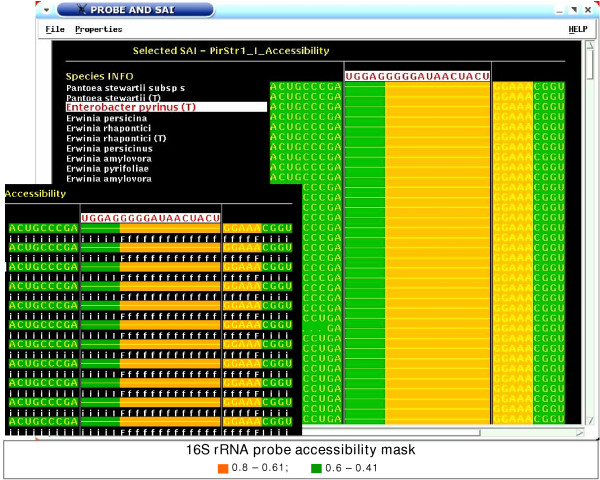
**Probe visualization window**. Probe visualization window displaying local alignments of probe target regions for members of the target group. Column statistics performed on the sequence alignments and structure masks are visualized in different background colors in the probe visualization window. This screenshot shows 16S rRNA accessibility map, where the experimentally determined relative fluorescence intensities are visualized in different colors (orange (0.8 – 0.61); green (0.6 – 0.41) [11]).

Visualization of probe candidates in a local alignment along with additional sequence associated information (SAI) can be managed with the PROBE Match SAI window. The neighboring region up to nine nucleotides on either terminus of the potential probe target is retrieved from the database. A local alignment of the extracted rRNA sequence is established and displayed along with the respective unique identifier such as ARB short_name, accession number, or any other underlying database fields (eg., Full Name, Group) (Figure 2, 3, 4). The user can select any information that is associated with the sequences (SAI) such as secondary structure masks (Figure 2) or any statistical calculations performed on the sequence level like sequence consensus, positional variability using parsimony method (Figure 3) or any other user defined models, filters or statistics as well as *in situ *accessibility maps for visualization (Figure 4). Different background colors can be assigned to characters and values or character groups and ranges of values of the particular SAIs, respectively. Optionally, the real characters or values contained in such SAIs can directly be visualized below the individual sequences. This offers a researcher a deeper insight in to the proposed oligonucleotide probe targets for careful examination of probe candidates *in silico *before making any decision on the selection of probe.

### Visualization of SAI in ARB primary structure editor

**Figure 5:**
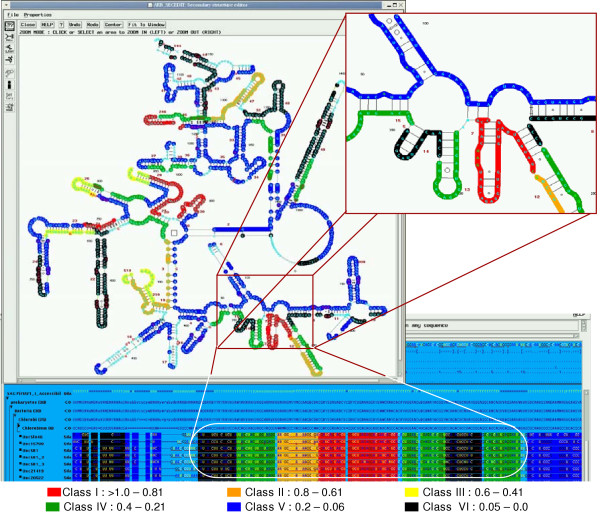
**ARB Primary and secondary structure windows**. ARB primary and secondary structure windows showing the distribution of relative fluorescence intensities of oligonucleotide probes targeting 16S rRNA structure of *M. sedula *[11]. The different background colors indicate brightness range of different classes (classes I through VI) with respect to the observed fluorescence intensities. Numbers displayed in black and lower case denotes the respective nucleotide positions and the numbers showing in red represent helix numbers in the model.

On the global alignment level, the user selected oligonucleotide probe is visualized in different background colors in the primary structure editor window of ARB [3]. The primary structure editor (Figure 5) of ARB displays multiple sequence alignments generated by the respective ARB software tools [3] of the selected sequences from the underlying database in the user-defined colors and symbols.

As already described for the local alignment level, any type of SAI can be visualized by the user defined background colors for the individual alignment columns. Customized color selections can be assigned to the different types of SAIs mentioned before. By scrolling the mouse or the use of ARB search tools, the user gets an easy access to the information for any range or the selection of sequences. In the context of probe evaluation for *in situ *hybridization experiments, mapping of experimentally derived *in situ *accessibility patterns onto the primary structures of interest certainly provides valuable support to the users for probe evaluation. Part of a multiple 16S rRNA sequence alignment is shown in the figure 5. The brightness classes defined for 16S rRNA structural model of *Methanosaeta sedula *[11] are mapped on the aligned sequences and indicated by background colors according to Behrens *et al *[11].

### Visualisation of SAI in ARB secondary structure editor

Theoretically as well as experimentally derived secondary structure information of SSU rRNA [15-17] is used more profoundly in sequence alignment refinement and probe design and evaluation. The tertiary structure of the SSU rRNA of the bacterium *Thermus thermophilus *which had been elucidated with atomic resolution by X-ray diffraction crystallography of ribosomal subunit [17] allows evaluating the exactness of the secondary structure model. The secondary structure of SSU rRNA has a crucial role in evaluating the proposed probe candidates prior to the actual experimentation. The ARB Secondary structure editor (Figure 5) provides the user with more intuitive graphical display of the secondary structure model of SSU rRNA. The user can visualize the entire SSU rRNA sequence of any organism in the respective database which fits into the common consensus model. The localization of proposed oligonucleotide probe targets can be visualized in customizable background colors.

## Conclusion

The evaluation of proposed probe target position with respect to higher-order rRNA structure is of more importance especially when probes are intended to be used for *in situ *hybridizations [7-12]. Albeit there have been several software programs developed for the design of rRNA targeted oligonucleotide probes [18,19], the criteria taken to design the probes are generally restricted to certain parameters such as size, nucleotide composition, specificity definition, and the general hybridisation behavior. None of the software described [18,19] takes into account the special requirements of rRNA targeted probes that are destined for FISH applications which is, the structure dependant probe accessibility of the ribosomal RNA. This feature has been developed and implemented in ARB. Using this tool, *in silico *probe design and evaluation can be performed with respect to *in situ *probe accessibility data. By identifying and excluding the probes targeting sites with a poor accessibility the number of time consuming empirical tests can be reduced.

## Availability and requirements

The entire ARB software and the periodic updates of well aligned and annotated ribosomal RNA databases are made freely available for the scientific community via World Wide Web [13]. Currently, the ARB Software is available for PCs running LINUX operating systems and SUN SOLARIS systems.

## Authors' contributions

YK developed and implemented the tool and drafted the manuscript. RW participated in design and implementation. SB and BF provided the accessibility data and revised the manuscript. FOG, RA and HM critically revised the manuscript. WL initiated the development of the tool and supervised the ARB project.
